# Detection of a G-Quadruplex as a Regulatory Element in *Thymidylate synthase* for Gene Silencing Using Polypurine Reverse Hoogsteen Hairpins

**DOI:** 10.3390/ijms21145028

**Published:** 2020-07-16

**Authors:** Eva Aubets, Alex J. Félix, Miguel Garavís, Laura Reyes, Anna Aviñó, Ramón Eritja, Carlos J. Ciudad, Véronique Noé

**Affiliations:** 1 Department of Biochemistry and Physiology, School of Pharmacy and Food sciences, and IN2UB, University of Barcelona, 08028 Barcelona, Spain; eaubets@ub.edu (E.A.); alexjimenezf@ub.edu (A.J.F.); cciudad@ub.edu (C.J.C.); 2 Instituto de Química y Física ‘Rocasolano’, CSIC, Serrano 119, 28006 Madrid, Spain; mgaravis@gmail.com; 3 Department of Chemistry Pulp and Paper Building, McGill University, 3420 Rue University, Montréal, QC H3A 0C7, Canada; 4 Institute for Advanced Chemistry of Catalonia (IQAC), CSIC, Networking Center on Bioengineering, Biomaterials and Biomedicine (CIBER-BBN), 08034 Barcelona, Spain; laura.reyes.fraile@gmail.com (L.R.); aaagma@cid.csic.es (A.A.); recgma@cid.csic.es (R.E.)

**Keywords:** G-quadruplex, PPRH, thymidylate synthase, anti-tumor therapy, 5-fluorouracil, gene silencing, gene regulation

## Abstract

Thymidylate synthase (TYMS) enzyme is an anti-cancer target given its role in DNA biosynthesis. TYMS inhibitors (e.g., 5-Fluorouracil) can lead to drug resistance through an autoregulatory mechanism of TYMS that causes its overexpression. Since G-quadruplexes (G4) can modulate gene expression, we searched for putative G4 forming sequences (G4FS) in the *TYMS* gene that could be targeted using polypurine reverse Hoogsteen hairpins (PPRH). G4 structures in the *TYMS* gene were detected using the quadruplex forming G-rich sequences mapper and confirmed through spectroscopic approaches such as circular dichroism and NMR using synthetic oligonucleotides. Interactions between G4FS and TYMS protein or G4FS and a PPRH targeting this sequence (HpTYMS-G4-T) were studied by EMSA and thioflavin T staining. We identified a G4FS in the 5’UTR of the *TYMS* gene in both DNA and RNA capable of interacting with TYMS protein. The PPRH binds to its corresponding target dsDNA, promoting G4 formation. In cancer cells, HpTYMG-G4-T decreased TYMS mRNA and protein levels, leading to cell death, and showed a synergic effect when combined with 5-fluorouracil. These results reveal the presence of a G4 motif in the *TYMS* gene, probably involved in the autoregulation of TYMS expression, and the therapeutic potential of a PPRH targeted to the G4FS.

## 1. Introduction

Thymidylate synthase (TYMS) has been widely studied as an anti-cancer target due to its essential role in the de novo synthesis of 2’-deoxythymidine-5’-monophosphate (dTMP), a critical precursor for DNA biosynthesis. TYMS catalyzes the conversion of 2’-deoxyuridine 5’-monophosphate (dUMP) to dTMP by transferring the methyl group from the 5,10-methylenetetrahydrofolate molecule (mTHF). Then, dTMP is successively phosphorylated to form 2’-deoxythymidine-5’-triphosphate (dTTP), generating one of the four fundamental nucleoside triphosphates involved in DNA synthesis [1,2]. 

TYMS inhibition affects highly proliferative cells (e.g., cancer cells) and has been established as a classic chemotherapeutic treatment during the last few decades [3]. Since its development in 1957, the nucleobase 5-fluorouracil (5-FU) has become one of the most widely used TYMS inhibitors for the treatment of different types of cancer, either as a single agent or in combination with other chemotherapeutics [2,4]. Intracellularly, 5-FU is converted to 5-fluoro-2’-deoxyuridine-5’-monophosphate (FdUMP), which is an analog of the endogenous ligand dUMP and binds to the nucleotide-binding domain of TYMS. This FdUMP-TYMS-mTHF ternary complex inhibits TYMS activity and leads to the suppression of dTMP synthesis, thus producing deoxynucleotide pool imbalance, increased levels of deoxyuridine triphosphate (dUTP) and, ultimately, DNA damage [4,5]. Nevertheless, the effectiveness of fluoropyrimidines or other TYMS inhibitors is compromised by the development of drug resistance. Some of these have been associated with high TYMS protein levels and are thought to be related to an autoregulatory mechanism of TYMS protein that modulates its own expression [6,7].

It has been proposed that the ligand-free TYMS protein is capable of binding to its own mRNA and represses its own translation. Two mRNA binding sites for TYMS have been identified so far in its mRNA: one located in the 5’ untranslated region (5’UTR), containing the translational start site (binding site I), and the other in the mRNA-coding region (binding site II) [8,9]. When TYMS protein is bound to dUMP or TYMS inhibitors such as fluoropyrimidines or antifolates [10], it cannot bind to its own mRNA and TYMS mRNA translation is not inhibited. This mechanism of translational regulation would explain the increased TYMS protein levels observed in tumor resistance cells [8,10,11].

Different strategies to overcome drug resistance have been developed, including antisense oligodeoxynucleotides (ASO) targeting different TYMS mRNA regions such as the protein binding site I, which might be an important regulator of translation [10]. The goal in the present work was to target other regulatory elements located in the *TYMS* gene, such as G-quadruplex structures (G4s). In the last few decades, the interest in G4s as gene regulation elements for anti-tumor applications has increased considerably [12]. G4s are nucleic acid secondary structures formed by guanine-rich RNA or DNA sequences whose basic structural unit is called G-tetrad, a square-planar arrangement of four guanines held together through Hoogsteen type associations. [13,14]. Stacking a minimum of two of these G-tetrads produces the four-stranded G4 structure that is further stabilized by monovalent cations (especially K^+^) and presents a high thermodynamic stability under physiological conditions [15]. G4s may have an important role in controlling different biological processes such as DNA replication [16], telomere maintenance [17] and mRNA transcription, processing and translation [18,19]. For this reason, G4s are mainly found in regulatory regions such as promoters, 5’UTRs, splicing sites and telomeres [20]. 

Here, we targeted a G4 forming sequence (G4FS) in the 5’UTR of the *TYMS* gene using a gene silencing tool developed in our laboratory named polypurine reverse Hoogsteen (PPRH) hairpins [21]. These molecules are non-modified single-stranded oligodeoxynucleotides formed by two antiparallel polypurine mirror repeat domains linked by a five-thymidine loop (5T). The intramolecular linkage consists of reverse Hoogsteen bonds between the purines, forming the hairpin structure. PPRHs can bind in a sequence-specific manner to polypyrimidine targets in the double-stranded DNA (dsDNA) via Watson–Crick bonds, thus producing a triplex structure and displacing the fourth strand of the dsDNA. This local distortion of the dsDNA leads to a transcriptional disruption that provokes the knockdown of the targeted gene [22]. Therefore, it is essential for PPRH design to find polypyrimidine tracts within the target gene sequence, which are mainly present in promoter or intronic regions [23]. During the last decade, we have used PPRHs as gene silencing tools for anti-cancer therapy [22,24,25,26,27], immunotherapy approaches [28,29,30] and targeting genes involved in resistance to chemotherapeutic drugs like methotrexate [31]. 

In this work, we identified and validated a G4 structure in the *TYMS* gene that can be targeted by a PPRH as a new approach to down-regulate TYMS expression. Treatment with this DNA hairpin was very effective against human cancer cells, and it acted synergistically when administered together with 5-FU. Additionally, we aimed to study the role of this G4 structure in the modulation of TYMS expression.

## 2. Results

### 2.1. Detection of a G4 Structure in the 5’UTR of TYMS

**Figure 1:**
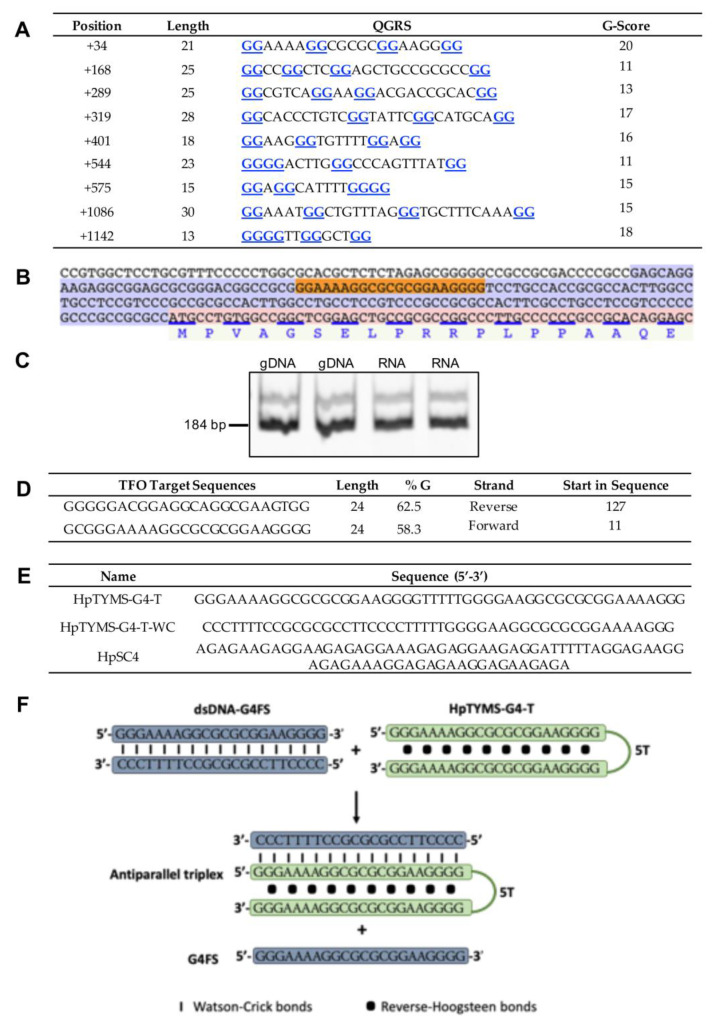
Identification of a G4FS in the TYMS gene and design of a PPRH targeting this site. (**A**) Putative G4-forming sequences detected in the TYMS gene using the QGRS mapper. The positions of the identified G4FS are referred to as the transcription start site considering the TYMS mRNA sequence NM_001071.3. The blue underlined Guanines (Gs) represent the ones implicated in G4 formation. (**B**) Localization of the G4FS with the higher G-score in the TYMS gene sequence (NM_001071.3). The orange highlighted sequence corresponds to G4FS. (**C**) The G4FS is located in the 5’UTR of the TYMS gene. PCR products (184 bp) obtained after amplification with primers 5’UTR-TYMS-Fw and 5’UTR-TYMS-Rv of both gDNA and cDNA/RNA samples. PCR products were resolved in a 6% polyacrylamide gel electrophoresis. (**D**) The first two polypurine sequences found in TYMS mRNA using the TFO searching tool. (**E**) Sequence of the specific PPRH targeting the G4FS (HpTYMS-G4-T), its corresponding Watson–Crick negative control (HpTYMS-G4-T-WC) and a scramble negative control (HpSC4). (**F**) Scheme showing the potential strand displacement produced when the PPRH (HpTYMS-G4-T) is mixed with dsDNA-G4FS. The formation of the antiparallel triplex by binding of the PPRH to the polypyrimidine oligonucleotide dissociates the polypurine oligonucleotide.

We searched G4FSs that could modulate TYMS expression using the quadruplex forming G-rich sequences (QGRS) mapper (Figure 1A). The sequence with the highest score (G20) was found in the 5’UTR of this gene (Figure 1B), according to reference [32] and in agreement with the human TYMS mRNA sequence NM_001071.3. However, in the last sequence version available in the NCBI gene database (NM_001071.4), the 5’UTR has been shortened (−69 nt) and the G4FS is excluded from the 5’UTR. Therefore, we carried out PCR reactions from either genomic or reverse transcribed DNA in order to test whether this G4FS was positioned in this untranslated region. Both genomic DNA (gDNA) and cDNA samples originated a main product of 184 bp (Figure 1C), thus confirming that the identified G4FS was actually located within the 5’UTR of the *TYMS* gene and not in the promoter. Moreover, we confirmed by sequencing that the 184 bp PCR product amplified from the cDNA sample corresponded to the 5’UTR sequence containing the G4FS. The human TYMS mRNA sequence (NM_001071.3) was subjected to the Triplex-Forming Oligonucleotide (TFO) searching tool [33]. The output (Figure 1D) was confronted with the list obtained with the QGRS mapper (Figure 1A). The matching sequence was used to design the PPRH targeting the G4FS (HpTYMS-G4-T) to decrease TYMS expression (Figure 1E). The mechanism of action of HpTYMS-G4-T is depicted in Figure 1F.

### 2.2. G4 Structure Confirmation in Both RNA and DNA

Next, we proceeded to confirm whether the G4FS (ssDNA-G4-Fw and RNA-G4) could form a G4 structure using different spectroscopic approaches such as circular dichroism (CD), UV absorbance spectroscopy (UV) and fluorescence and nuclear magnetic resonance (NMR) using synthetic oligonucleotides. UV absorbance and CD spectroscopies have played important roles in the verification of G-quadruplex folding [34,35]. Denaturation or melting curves recorded at 295 nm are commonly used to determine the thermodynamic properties of G-quadruplex [34]. In addition, a thermal difference spectrum (TDS) (absorption difference between unfolded and folded form) is applied in order to confirm the existence of G-quadruplex structures and differentiate them from other DNA structures such as duplexes or triplexes [36].

Oligonucleotides ssDNA-G4-Fw and RNA-G4 (Table 1) contain the G4FS identified by the QGRS mapper (Figure 1A,B) in both DNA and RNA backbones. The melting curves of these oligonucleotides were recorded in two different buffers. One of them, K^+^ solution, contains a relatively large amount of potassium, which has been described to stabilize the G-quadruplex structure. The other is a phosphate-buffered solution (PBS) containing sodium cations. The thermal denaturation profiles exhibited hypochromism at 295 nm with increasing temperature, indicating the denaturation of a potential G-quadruplex structure. Both denaturation curves showed similar melting profiles with similar melting temperatures, being 37 °C in K^+^ solution, which is slightly higher than in PBS (35 °C) for ssDNA-G4-Fw. RNA-G4 showed similar behavior, with increased stability compared to ssDNA-G4-Fw, 47 °C in K^+^ buffer and 40 °C in PBS. TDS, especially in K^+^ buffer, showed the spectra profile assigned to a G-quadruplex with maxima around 243 and 273 and minima at around 295 nm [36] (Figure S1). 

**Figure 2:**
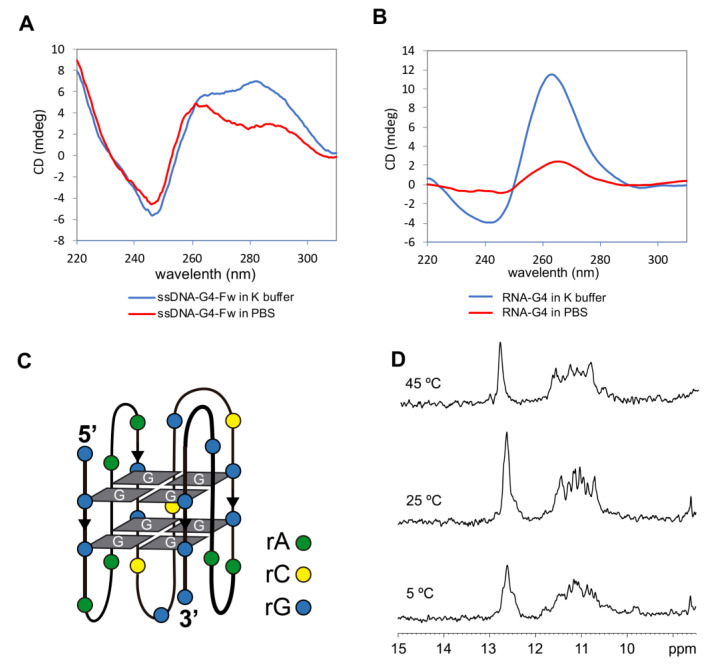
Structural characterization of G4FS in both RNA and DNA oligonucleotides. CD spectra of ssDNA-G4-Fw (**A**) and RNA-G4 (**B**) in K^+^ buffer (blue line) and PBS (red line) at 20 °C, showing a shape consistent with the formation of G-quadruplex structures. (**C**) Illustration representing the G-quadruplex folding of the G4-RNA sequence. Each color dot represents a ribonucleotide: adenine (rA), citosine (rC) and guanine (rG). (**D**) Imino region of monodimensional ^1^H-NMR spectra of ssDNA-G4-Fw in H_2_O/D_2_O (90:10) at different temperatures. The spectra show signals between 10 and 12 ppm, characteristic of imino protons taking part in G-tetrads. Buffer conditions: 25 mM KPi pH 7, 100 mM KCl.

CD spectra were recorded using the same buffers (Figure 2A,B). Spectra were indicative of hybrid G-quadruplex, with positive bands at ~260 and 295 nm and a negative band at 245 nm for ssDNA-G4-Fw (Figure 2A), and a parallel G-quadruplex structure with positive bands ~260 and a negative band at 245 nm for RNA-G4 (Figure 2B). A schematic illustration of the possible G-quadruplex structure of RNA-G4 is shown in Figure 2C.

The imino region of the one-dimensional ^1^H-NMR spectrum (Figure 2D) of ssDNA-G4-Fw showed signals between 10 and 12 ppm, which are within the range of the chemical shifts of guanine imino protons involved in G-tetrads. The signals observed between 12 and 13 ppm belong to imino protons forming GC Watson–Crick base pairs. Most probably, these signals are due to the formation of G:C:G:C tetrads within the G-quadruplex. Such tetrads, resulting from the association of two G:C base pairs, are not unusual and accommodate well between G:G:G:G tetrads [37]. All the imino signals remained visible at 45 °C (Figure 2D), indicating the remarkable thermal stability of the structure, which is frequent for G-quadruplexes [15]. 

Thioflavin T (ThT) is an efficient fluorescent sensor of G-quadruplex and it has been used to discriminate G-quadruplex structures from other structures [38]. To this end, we carried out fluorescence spectra titrations by adding the oligonucleotide (ODN) dissolved in PBS to a solution of ThT. We observed an increase in fluorescence with increasing concentrations of the ODN, which enables us to determine an association constant following the method described in the literature [39]. The association constants for ssDNA-G4-Fw were found to be 2.72 × 10^6^ and 2.90 × 10^6^ for RNA-G4, both in the range of the values described for other G-quadruplexes [38] (Figure S2).

### 2.3. TYMS Protein Binds to the G4FS

To study the possible binding of the TYMS protein to the G4FS contained in the 5’UTR of the *TYMS* gene, we performed different electrophoretic mobility shift assays (EMSA) using radioactively labeled ssDNA-G4-Fw-E or RNA-G4 oligonucleotides (Table 1).

**Figure 3:**
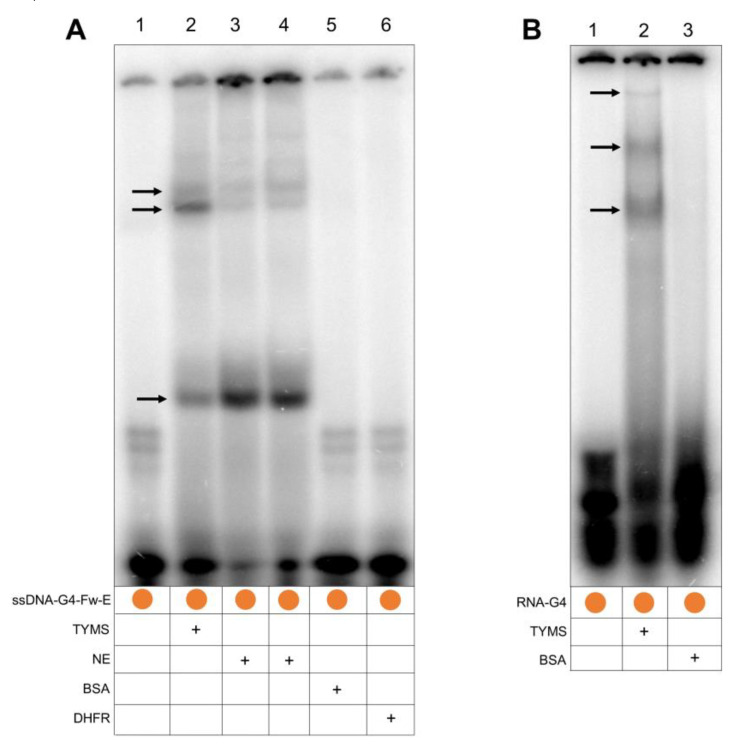
Binding of TYMS protein to the G4FS mRNA. EMSAs using ^32^P-radiolabeled ssDNA (**A**) or RNA (**B**) probes corresponding to the G4FS. (**A**) Lane 1, ssDNA-G4-Fw-E probe alone; lane 2, ssDNA-G4-Fw-E plus TYMS protein (1.5 µg); lane 3 and 4, ssDNA-G4-Fw-E plus nuclear protein extract (2 µg) in duplicate; lane 5, ssDNA-G4-Fw-E plus BSA protein (2 µg); lane 6, ssDNA-G4-Fw-E plus DHFR protein (2 µg). (**B**) Lane 1, RNA-G4 probe alone; lane 2, RNA-G4 plus TYMS protein (1.5 µg); lane 3, RNA-G4 plus BSA protein (2 µg). Arrows indicate the shifted bands corresponding to the different molecular species.

In the case of ssDNA-G4-Fw-E, we observed three shifted bands—two with high molecular weight—when incubating the probe with purified TYMS protein (Figure 3A, lane 2). In a similar way, incubation of a nuclear extract from cervical carcinoma cells (HeLa) with the probe produced the same shifted bands (Figure 3A, lane 3-4) that could correspond to the binding of the TYMS protein present in the nuclear extract. In contrast, the incubation of the ssDNA-G4-Fw-E probe with either bovine serum albumin (BSA) or dihydrofolate reductase (DHFR) proteins as negative controls did not show any shifted bands (Figure 3A, lane 5-6). Therefore, TYMS protein specifically binds to the G4FS located in the 5’UTR of the *TYMS* gene. 

Since we observed the binding of the TYMS protein to the ssDNA-G4-Fw-E probe, we checked whether this binding could occur using the RNA sequence corresponding to the same G4FS. The incubation of TYMS protein with the RNA probe (RNA-G4) showed three shifted bands (Figure 3B, lane 2), thus demonstrating that TYMS protein binds to this particular G4FS located in the 5’UTR of its own mRNA. The incubation of the probe with the BSA protein did not produce any binding (Figure 3B, lane 3).

### 2.4. TYMS Protein and HpTYMS-G4-T Compete to Bind to the G4FS in the dsDNA

**Figure 4:**
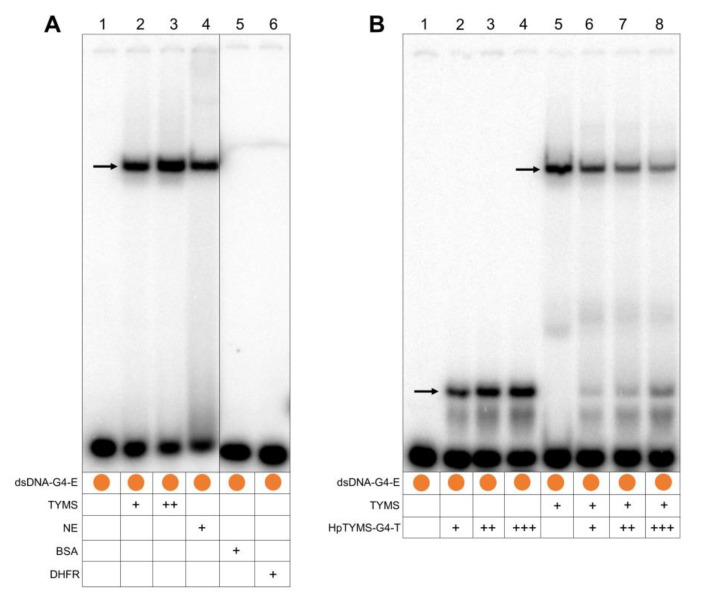
Binding of TYMS protein to the ^32^P-radiolabeled dsDNA probe corresponding to the G4FS. (**A**) Lane 1, dsDNA-G4-E probe alone; lane 2, dsDNA-G4-E plus TYMS protein (1.5 µg); lane 3, dsDNA-G4 plus TYMS protein (3 µg); lane 4, dsDNA-G4-E plus HeLa nuclear protein extract (NE) (2 µg); lane 5, dsDNA-G4-E plus BSA protein (2 µg); lane 6, dsDNA-G4-E plus DHFR protein (2 µg). (**B**) Competition assay between the TYMS protein and the HpTYMS-G4-T for the binding to the G4FS in the dsDNA. Lane 1, dsDNA-G4-E probe alone; lane 2, dsDNA-G4-E plus HpTYMS-G4-T (10 ng); lane 3, dsDNA-G4-E plus HpTYMS-G4-T (30 ng); lane 4, dsDNA-G4-E plus HpTYMS-G4-T (60 ng); lane 5, dsDNA-G4-E plus TYMS protein (1.5 µg); lane 6, dsDNA-G4-E plus TYMS protein (1.5 µg) competed with HpTYMS-G4-T (10 ng); lane 7, dsDNA-G4-E plus TYMS protein (1.5 µg) competed with HpTYMS-G4-T (30 ng); lane 8, dsDNA-G4-E plus TYMS protein (1.5 µg) competed with HpTYMS-G4-T (60 ng). Arrows indicate the shifted bands corresponding to the different molecular species.

Since TYMS protein can also be found in the cell nucleus, we explored the possible binding of this protein to the dsDNA corresponding to the G4FS. In these experiments, we observed a unique shifted band when incubating the dsDNA-G4-E probe (prepared by hybridization of ssDNA-G4-Fw-E and ssDNA-G4-Rv-E, Table 1) with TYMS protein (Figure 4A, lane 2). The intensity of this shifted band was increased when a higher amount of TYMS protein was added to the reaction (Figure 4A, lane 3). Additionally, the incubation of the probe with a HeLa nuclear extract (2 µg) produced a shifted band with the same mobility as the one obtained with purified TYMS protein (Figure 4A, lane 4). We confirmed that the binding of TYMS protein to the G4FS was specific since incubation with BSA or DHFR proteins did not produce any shifted bands (Figure 4A, lane 5–6). 

Next, we designed the PPRH sequence (HpTYMS-G4-T) against the homopyrimidine complementary sequence of G4FS (Figure 1E). Then, we demonstrated that this PPRH oligonucleotide was able to bind the dsDNA-G4-E probe, observing one shifted band whose intensity increased depending on the amount of HpTYMS-G4-T added to the reaction (10, 30 or 60 ng) (Figure 4B, lane 2–4). We also performed a competition assay between HpTYMS-G4-T and TYMS protein. To do so, different amounts of HpTYMS-G4-T (10, 30 and 60 ng) were added to the binding reaction, together with a fixed amount of TYMS protein, showing a decrease in the intensity of the shifted band, corresponding to the binding of TYMS protein to the probe (Figure 4B, lane 5–8). Interestingly, the shifted band corresponding to the binding of HpTYMS-G4-T to the probe reappeared, and its intensity was proportional to the amount of HpTYMS-G4-T added to the reaction (Figure 4B, lane 5–8). Therefore, we demonstrated that HpTYMS-G4-T and TYMS protein bind to the same G4FS site in the dsDNA.

### 2.5. HpTYMS-G4-T Promotes the Formation of the G4 Structure

**Figure 5:**
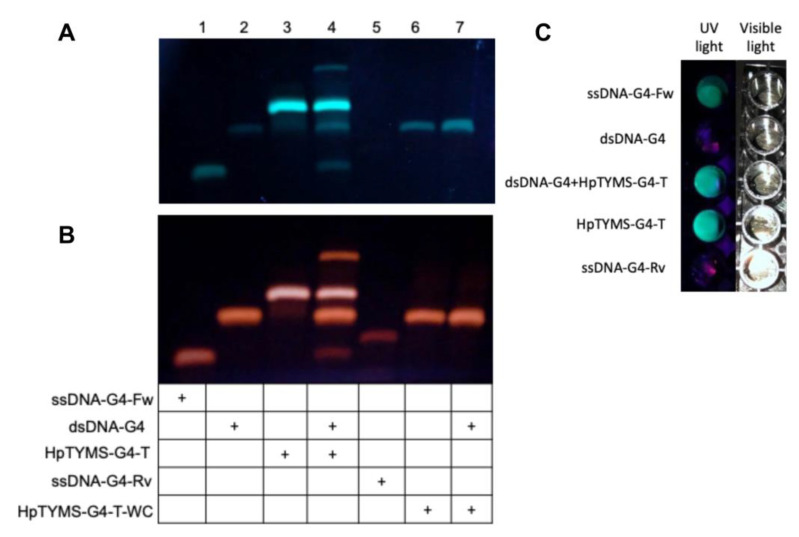
DNA binding assays and G4 structure detection using ThT. Samples incubated at 90 °C were cooled down slowly to room temperature and loaded in a nondenaturing 12% acrylamide gel supplemented with 10 mM KCl. The gel was visualized under UV light after ThT staining (**A**) and EthBr staining. (**B**) Lane 1, ssDNA-G4-Fw alone; lane 2, dsDNA-G4 alone; lane 3, HpTYMS-G4-T alone; lane 4, dsDNA-G4-2 plus HpTYMS-G4-T; lane 5, ssDNA-G4-Rv alone; lane 6, HpTYMS-G4-T-WC alone; lane 7, dsDNA-G4 plus HpTYMS-G4-T-WC. (**C**) G4 detection in samples directly incubated with ThT and visualized under UV light. Samples were prepared as indicated in Section 4.11 of Materials and Methods but containing 1 μM of each oligonucleotide. When samples reached room temperature, 8 μM of ThT was added and samples were visualized under UV light lamp or visible light.

**Figure 6:**
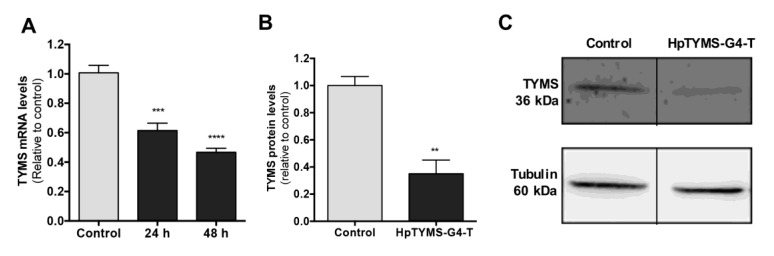
Effect of HpTYMS-G4-T on the levels of TYMS mRNA and protein. Hela cells (30,000) were incubated with 100 nM of HpTYMS-G4-T. (**A**) TYMS mRNA levels 24 h and 48 h after transfection; RNA levels determined by RT-qPCR. Cyclophilin B (PPIB) was used to normalize the results. Statistical significance was determined using one-way ANOVA with Dunnett’s multiple comparisons test (*** *p* < 0.001, **** *p* < 0.0001). (**B**) TYMS protein levels in HeLa cells after 24 h of transfection. Statistical significance was determined using an unpaired Student’s T test (** *p* < 0.01). Tubulin protein levels were used to normalize the results. (**C**) Representative images of Western blots.

HpTYMS-G4-T was designed to bind to its polypyrimidine target sequence in the dsDNA and form a DNA triplex. This would displace the G-rich sequence of the duplex that consequently could fold into a G4 structure (Figure 1F). To verify this possibility, the products of different binding assays were separated on a nondenaturing gel (Figure 5), which was then stained with ThT to detect the G4 structures formed [40]. The ssDNA-G4-Fw sequence was stained by ThT, as expected for a G4 structure (Figure 5A, lane 1), while dsDNA-G4 (hybrid from ssDNA-G4-Fw and ssDNA-G4-Rv) did not (Figure 5A, lane 2). The PPRH HpTYMS-G4-T was also detected by ThT staining, which is consistent with the fact that its sequence contains two repeats of G4-Fw and thus is able to fold into G4 structures. The PPRH samples showed a strong band and a shadow since they can adopt different conformations [24,41] (Figure 5A, lane 3). Incubation of dsDNA-G4 concurrently with the PPRH led to the formation of two extra bands, the upper corresponding to the ssDNA-G4-Rv/PPRH DNA triplex and the lower corresponding to the ssDNA-G4-Fw strand displaced from dsDNA-G4, which did form a G4 structure (Figure 5A, lane 4). As a negative control, we incubated ssDNA-G4-Rv, which is not a G4FS and, consequently, was not stained with ThT (Figure 5A, Lane 5). In addition, we used HpTYMS-G4-T-WC, a hairpin intramolecularly bound by Watson–Crick base pairs and thus not able to produce the DNA triplex and to displace the fourth strand. Accordingly, we did not observe the formation of the triplex between HpTYMS-G4-T-WC and the dsDNA-G4 (Figure 5A, lane 7). However, since one of the domains of HpTYMS-G4-T-WC still contains a G4FS, the oligonucleotide was also stained by ThT (Figure 6A, lane 6). Then, after staining the same gel with ethidium bromide (EthBr), the species corresponding to dsDNA-G4 (Figure 5B, lane 2, lane 4 and lane 7) and ssDNA-G4-Rv (Figure 5B, lane 5) were visualized. G4 structures were also detected when ThT was directly added to the different binding assays incubations instead of electrophoresing the samples and staining the gel afterwards (Figure 5C). 

### 2.6. Effect of HpTYMS-G4-T on the Levels of TYMS mRNA and Protein

To confirm a specific decrease in *TYMS* gene expression, TYMS mRNA levels in cervical carcinoma cells (HeLa) treated with HpTYMS-G4-T for 24 h or 48 h were analyzed. Cells incubated with HpTYMS-G4-T showed a decrease in *TYMS* gene expression, both at 24 h and 48 h (1.6-2.1-fold decrease relative to control) (Figure 6A). In addition, a 65% decrease in TYMS protein levels was observed after 24 h of incubation with the HpTYMS-G4-T (Figure 6B,C). 

### 2.7. Effect of HpTYMS-G4-T as a Single Agent or Combined with 5-FU on HeLa Cell Viability

**Figure 7:**
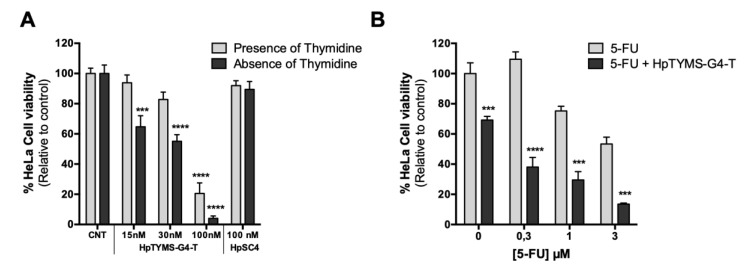
Effect on HeLa cell viability of HpTYMS-G4-T. (**A**) Dose–response of HeLa cell viability upon treatment with the HpTYMS-G4 and the HpSC4 in the absence (RPMI medium) or presence of thymidine (F12 medium). (**B**) Effect on cell viability when combining HpTYMS-G4-T with 5-FU. HeLa cells were treated with 5-FU at different concentrations (0.3, 1, 3 µM) in the absence or presence of HpTYMS-G4-T (15 nM). Statistical significance was determined using two-way ANOVA with Sidak’s multiple comparisons test (*** *p* < 0.001, **** *p* < 0.0001). When combining the PPRH with 5-FU, statistical significance was estimated by comparing the effect of the PPRH alone to the control or comparing the effect of 5-FU plus PPRH to 5-FU alone. When transfecting the PPRH at 100 nM, 10 µM of DOTAP was used, whereas for 15 nM or 30 nM of PPRH, 5 µM DOTAP was employed.

Since TYMS is involved in the de novo synthesis of dTTP, inhibition of TYMS expression using HpTYMS-G4-T was expected to reduce the viability of cancer cells and to produce a higher effect when the incubation was carried out in thymidine deficient medium. Therefore, HpTYMS-G4-T was incubated either in the presence or in the absence of thymidine. The PPRH was cytotoxic in a dose-dependent manner in both media, whereas HpSC4 did not produce any effect on cell viability. The highest cytotoxicity was reached at 100 nM in the absence of thymidine, with less than 5% of the viable cells remaining (Figure 7A). Once the effectiveness of HpTYMS-G4-T was established, we studied the effects of combining this specific PPRH with 5-FU, a classic inhibitor of TYMS protein. The PPRH (15 nM) presented a synergic effect when combined with different doses of 5-FU (0.3, 1 and 3 μM), reducing cell viability down to 38%, 30%, and 14%, respectively (combination index calculated by the CompusSyn software [42]) (Figure 7B). 

As a control, the influence of 5-FU as a single agent on TYMS expression was also addressed. HeLa cells incubated for 24 h with 3 μM of 5-FU showed a slight increase in TYMS mRNA levels, which were re-established after 48 h. At the protein level, a three-fold increase in total TYMS protein was observed after 24 h (Figure S3). These results are in agreement with [43,44,45].

### 2.8. Effect of HpTYMS-G4-T on PC-3 Cell Viability and mRNA Levels

**Figure 8:**
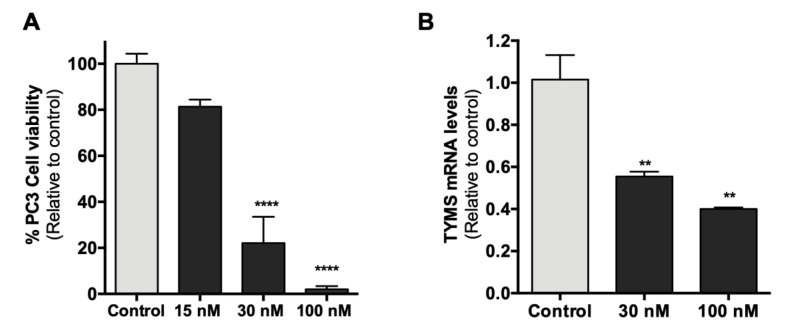
Effect of HpTYMS-G4-T on cell viability and TYMS mRNA levels in PC-3 cells. (**A**) Dose–response of PC-3 cell viability upon treatment of HpTYMS-G4-T in the presence of thymidine. (**B**) TYMS mRNA levels. After 24 h of transfection, RNA extraction was conducted and RNA levels determined by RT-qPCR. PPIB was used to normalize the results. Statistical significance was determined using one-way ANOVA with Dunnett’s multiple comparisons test (** *p* < 0.01, **** *p* < 0.0001).

We further examined whether the antiproliferative effect of HpTYMS-G4-T on HeLa cells was reproducible in other human cancer cell lines. HpTYMS-G4-T also produced a decrease in the viability of prostate adenocarcinoma cells (PC-3) in a dose–response manner, with only 4% of the viable cells remaining at a concentration of 100 nM of PPRH (Figure 8A). In addition, after 24 h of transfection, PC-3 cells treated with 30 nM or 100 nM of HpTYMS-G4-T showed a 45% and 60% decrease in TYMS mRNA levels relative to the control, respectively (Figure 8B). 

## 3. Discussion

In this work, we describe the effects of a PPRH targeting a polypyrimidine strand complementary to a newly identified G4 motif present in the 5’UTR of the *TYMS* gene, whose encoded protein is a classical anti-cancer target due to its role in DNA synthesis [2]. Since it is known that G4 structures can be important gene regulatory elements, we searched for G4 structures in the *TYMS* gene using the QGRS mapper, a computational tool for the prediction of quadruplex forming G-rich sequences [12,46]. The spectroscopic experiments carried out in this work confirmed that the G4FS detected in the 5’UTR of the TYMS gene by the QGRS mapper fold into a G4 structure as both RNA and DNA. 

Since there was a discrepancy in the extension of the 5’UTR sequence of the human TYMS mRNA in the last two accession numbers (NM_001071.4 and NM_001071.3) available in the NCBI database, we verified by PCR and RT-PCR that the G4FS identified in this work was actually located in the 5’UTR of the *TYMS* gene and not in its promoter sequence, in agreement with the original sequence for the *TYMS* gene reported in [32].

Interestingly, some metabolic enzymes have been described as multifunctional proteins performing distinct biochemical functions in the cytoplasm and nucleus, which are involved in transcriptional, posttranscriptional and translational regulation. Among them, we find glyceraldehyde 3-phosphate dehydrogenase, arg 5,6, enoyl-CoA hydratase, lactate dehydrogenase, threonyl-tRNA-synthetase, dihydrofolate reductase and Thymidylate synthase [47,48,49]. Moreover, it has been demonstrated that G4FS are putative protein binding regions that can act as regulatory elements of gene expression [12]. For these reasons, we aimed to study whether TYMS protein could directly interact with the G4FS present in its own 5’UTR (TYMS-G4FS). Since TYMS is a self-mRNA targeting protein binding to at least two different sites and inhibiting its own translation [8,9], we also evaluated the possible interaction between TYMS and the G4FS as mRNA species. This was confirmed by EMSA, thus proving that this protein has another binding site in its own mRNA that could also correspond to a translational regulator. 

In addition, we explored whether TYMS protein was also interacting with the dsDNA formed by the TYMS-G4FS and its complementary strand, as this interaction could be important for the regulation of gene expression at the transcriptional level. EMSAs showed the specific binding of TYMS protein to the G4FS site, both as dsDNA and ssDNA. The same G4FS site, through its polypyrimidine tract, was targeted by the binding of the designed PPRH (HpTYMS-G4-T), which caused a decrease in the expression of TYMS. These results suggest that TYMS protein could be involved in its own transcriptional regulation upon binding to this G4FS site.

Altogether, the observed interactions between TYMS protein and G4FS, either as mRNA or DNA, highlight that this site could be involved in translational and transcriptional regulation. Moreover, it has been previously demonstrated that C-MYC [50], p53 [51,52] and IFN-induced 15 kDa protein [53] mRNA translation is also controlled by TYMS, highlighting the important role of TYMS protein regulating not only its own expression but also that of other proteins.

Furthermore, in this work, we uncover the importance of the TYMS-G4FS region in the regulation of TYMS expression. Whether G4 structures promote or inhibit transcriptional or translational processes is a discrepant issue. While some studies indicate that G4s may positively contribute to gene expression through protein binding, others maintain that G4 structures may act as obstacles [54]. For instance, it has been reported that a G4 located in the *C-MYC* promoter interacts with the nucleic acid binding protein (CNBP) and NM23-H2, contributing to the enhancement of its own transcription [55]. In contrast, Kumari et al. reported that a G4 structure located in the 5’UTR of the NRAS mRNA inhibited its own translation [56]. This could also be the function of the G4FS identified in the 5’UTR of TYMS. One hypothesis is that the interaction that we observed between TYMS protein and the G4FS, either as DNA or RNA, could be preventing the function of the transcriptional or translational machineries. According to this premise, when TYMS is bound by its substrates (mTHF and dUMP) or by TYMS inhibitors, it does not interact with G4FS, thus increasing TYMS expression and leading to resistance to inhibitors. This proposition is in concordance with the currently postulated TYMS autoregulatory mechanism [8,9], but with an additional self-mRNA binding site and extrapolating the effect at the transcriptional level.

In view of the relevant role of G4 motifs in the regulation of gene expression, a wide range of G4 ligands have been developed for anti-tumor applications, such as ligands that stabilize G4 structures in the telomere region to block telomerase activity [57,58] and those targeting G4 structures located in promoters, 5’UTRs or 3’UTRs to regulate different cancer-related genes [12,59,60,61,62]. Several G4 ligands have demonstrated their potential in vivo [63] and some have reached clinical trials [64,65,66,67]. However, one of the main problems with these compounds is their lack of specificity and the possible off-target effects due to the high number of putative G4 structures present in the human genome [20,68].

In contrast, PPRHs can be designed to bind specifically to a polypyrimidine sequence that is complementary to a given G4FS. In fact, we proved that HpTYMS-G4-T binds in a specific manner to its target sequence, located in the complementary polypyrimidine strand of G4FS. Accordingly, our binding assays with ThT staining showed that even in conditions in which a PPRH can fold into a G4 structure, this does not impair its binding to the dsDNA, thus confirming our previous data [41]. In addition, dsDNA/PPRH triplex formation promoted the displacement of the polypurine strand and its folding into a G4 structure, thus the PPRH may contribute to stabilizing the formation of the G4 structure and would regulate transcription of this gene. We also corroborated the notion that Hoogsteen bonds stabilizing the PPRH are essential for triplex formation, since the Watson–Crick hairpin HpTYMS-G4-WC was not able to form the triplex.

One of the goals of this work was to demonstrate the effect of HpTYMS-G4-T on cancer cells. In this regard, we demonstrated that the PPRH targeting G4FS decreased both TYMS mRNA and protein levels in a specific manner. This down-regulation of TYMS expression led to a decrease in cell viability in a dose-dependent manner in both HeLa and PC3 cancer cells. It is worth noting that the cytotoxic effect of HpTYMS-G4-T was lower when cells were incubated in medium containing thymidine. Similarly, Schmitz et al. also reported that the effect of a siRNA targeting TYMS mRNA was reversed with 10 µM of thymidine [69]. These data suggest that the cytotoxic effect of HpTYMS-G4-T results from the inhibition of thymidylate biosynthesis, caused by the specific inhibition of TYMS expression. 

Other gene silencing molecules such as siRNAs [10,69], ASOs [70,71,72] or peptides [73] targeting TYMS mRNA also succeeded in down-regulating TYMS expression. However, PPRHs present some advantages compared to other silencing tools. PPRHs are nonmodified DNA molecules that inhibit gene expression at lower concentrations than those needed for ASOs [22], they are more economical, less immunogenic and have a longer half-life than siRNAs [74]. Recently, we also demonstrated in a pharmacogenomic study that PPRHs do not present hepatotoxicity or nephrotoxicity in vitro [75].

In addition, since TYMS’s autoregulatory translation mechanism has been associated with traditional TYMS inhibitor resistance [6,7,8], we evaluated the effect of HpTYMS-G4-T in combination with 5-FU. We demonstrated the synergic effect of HpTYMS-G4-T in HeLa cells treated concurrently with 5-FU. Our results are in accordance with others proving that strategies focused on reducing *TYMS* gene expression can sensitize cells to traditional TYMS inhibitors [69,72]. 

Overall, in this work, we identified and confirmed a G4 structure in the 5’UTR of the *TYMS* gene that could be involved in transcriptional and translational autoregulation of TYMS expression. We also showed that a PPRH designed against this G4FS presented therapeutic potential as a single agent or in combination with 5-FU treatment. Therefore, we provide new insights for the design of strategies that improve the effect of traditional TYMS inhibitors.

## 4. Materials and Methods 

### 4.1. Bioinformatic Detection of G4-Forming Sequences 

Putative G4FS were analyzed using the QGRS mapper (http://bioinformatics.ramapo.edu/QGRS/index.php). This software program generates information on the composition and distribution of putative QGRS in nucleotide sequences and is based on published algorithms for the recognition and mapping of putative QGRS [46].

### 4.2. Design of Polypurine Reverse Hoogsteen Hairpins 

The search of polypurine sequences that serve to design PPRH hairpins was performed using the Triplex-Forming Oligonucleotide Target Sequence Search software (http://utw10685.utweb.utexas.edu/tfo/ MD Anderson Cancer Center, University of Texas) [33]. The design consisted of two antiparallel mirror repeats of polypurine stretches, intramolecularly bound by reverse Hoogsteen bonds and linked by a loop of five thymidines (5T). As negative controls, we designed a hairpin with intramolecular Watson–Crick bonds instead of Hoogsteen bonds (HpTYMS-G4-T-WC) and a scrambled hairpin (HpSC4) (Figure 1E). The sequences were synthetized as non-modified oligodeoxynucleotides by Sigma-Aldrich (Haverhill, UK), resuspended in sterile Tris-EDTA buffer (1 mM EDTA and 10 mM Tris, pH 8.0) (Sigma-Aldrich, Madrid, Spain) and stored at −20 °C until use.

### 4.3. RNA and gDNA Extraction

Total RNA was extracted from cells using TRIzol® (Life Technologies, Barcelona, Spain), following the manufacturer’s specifications. RNA intended for PCR assays was treated with DNAse I, RNAse-free (ThermoFisher, Barcelona, Spain). Total gDNA was extracted using the Wizard genomic DNA purification kit (Promega, Madrid, Spain), following the manufacturer’s instructions. RNA and gDNA concentrations were determined by measuring its absorbance at 260 nm using a NanoDrop ND-1000 spectrophotometer (ThermoFisher, Barcelona, Spain).

### 4.4. Reverse Transcription

cDNA was synthesized in a 20 μL reaction mixture containing 1 μg of total RNA, 125 ng of random hexamers (Roche, Spain), 500 μM of each deoxynucleotide triphosphate (Bioline, Barcelona, Spain), 2 μL of buffer (10×), 20 units of RNAse inhibitor and 200 units of Moloney murine leukemia virus reverse transcriptase (Last three from Lucigen, WI, USA). The reaction was carried out at 42 °C for 1 h.

### 4.5. PCR

gDNA and cDNA samples from HeLa cells were used as templates to perform PCR reactions in order to locate sequences corresponding to the 5’UTR. Reactions were performed in a final volume of 50 µL containing either 500 ng of gDNA or 250 ng of cDNA, 5 μL of buffer 5×, 500 ng forward primer, 500 ng reverse primer, 200 μM dNTPs (Bioline, Barcelona, Spain), 1.25 U OneTaq DNA polymerase (New England Biolabs, Barcelona, Spain) and H_2_O mQ. Cycling conditions were as follows: 3 min denaturation at 94 °C, followed by 30 cycles of 30 s at 94 °C, 1 min at 59 °C and 1 min at 68 °C, and a final extension step of 5 min at 68 °C. PCR products were resolved in a nondenaturing 6% polyacrylamide gel electrophoresis in 1× TBE buffer. Gels were visualized using Gel Doc™ EZ and the Image Lab Software, Version 6.0 (Bio-Rad, Madrid, Spain). Forward (5’UTR-TYMS-Fw) and reverse primers (5’UTR-TYMS-Rv) were designed to hybridize to a sequence located 32 nt upstream and 109 nt downstream from the G4FS, respectively. The 5’UTR-TYMS-Rv primer covers 25 nt of the translating mRNA. The 184 bp PCR product was sequenced in Macrogen (Amsterdam, the Netherlands).

We confirmed that the DNAse digestion was completely achieved by performing a PCR with primers located in exon 1 (APRT-Ex1-Fw) and exon 2 (APRT-Ex2-Rv) of the human *APRT* gene, intervened by a short intron of 163 bp. The amplification of the gDNA sample led to a product of 291 bp (including intron 1), whereas in the case of the RNA (treated with DNAse), the product had a length of 128 bp (excluding intron 1). Primer sequences are shown in Table 1.

### 4.6. CD and UV Absorbance Spectroscopy

The CD spectra were recorded on a JASCO spectropolarimeter J-810 at 20 °C with a scanning speed of 100 nm/min, a response time of 4 s, 0.5 nm data pitch and 1 nm bandwidth. The samples (4 µM) were dissolved in the above buffers, annealed and slowly cooled to room temperature and left at 4 °C for at least one night. 

The thermal melting curves were obtained following the absorption change at 295 nm for the ODN from 15 °C to 70 °C, with a linear temperature ramp of 0.5°/min in Teflon-stoppered 1-cm path-length quartz cells, on a JASCO V-650 spectrophotometer equipped with a Peltier temperature control. UV spectra of the oligonucleotides were recorded at 15° (folded) and 80 °C (unfolded) to calculate the TDS. The measurements were conducted in either 10 mM sodium cacodylate buffer plus 100 mM KCl (pH 7.0) or PBS.

### 4.7. Thioflavin T Fluorescence Spectroscopy

ThT titrations were carried out with a JASCO FP-6200 spectrofluorometer with a temperature-controlled circulator JASCO ETC-272T. The fluorescence spectra were acquired using a quartz cuvette with a 10-mm path length. In the fluorescence measurements, both the excitation and emission slits were 10 nm, the excitation wavelength was set to 430 nm, and the scan speed was 250 nm/min. Fluorescence spectra were recorded between 450 to 650 nm. ThT (3 µM) was titrated with increasing concentrations of ODN (O-6 µM) to measure the binding constant. The fluorescence intensity at 491 nm was plotted as a function of the oligonucleotide concentration. The data were fitted according to a 1:1 binding model. 

### 4.8. Nuclear Magnetic Resonance

The sample for NMR was prepared by dissolving the oligonucleotide at 180 μM concentration in 25 mM K2HPO4; pH7, 100 mM KCl buffer containing 10% D_2_O. NMR spectra were acquired with a Bruker QANUC 800 MHz spectrometer equipped with a cryoprobe and processed with TOPSPIN 2.1 software. An excitation sculpting pulse program was used to supress the water signal while detecting rapid interchangeable imino protons.

### 4.9. RT-qPCR

To determine TYMS mRNA levels, a QuantStudio 3 Real-Time PCR System (Applied Biosystems, Barcelona, Spain) was used. The reaction was performed in a final volume of 20 μL, containing 1× SYBR Universal PCR Master mix (Applied Biosystems, Barcelona, Spain), 0.25 μM of reverse and forward primers (Sigma- Aldrich, Madrid, Spain), 3 μL of cDNA and H_2_O mQ. PCR cycling conditions were 10 min denaturation at 95 °C, followed by 40 cycles of 15 s at 95 °C and 1 min at 60 °C. The mRNA quantification was performed using the ΔΔCt method, where Ct is the threshold cycle that corresponds to the cycle where the amount of amplified mRNA reaches the threshold of fluorescence. Cyclophylin B (PPIB) was used as an endogenous control to normalize the results. Primer sequences for RT-qPCR are detailed in Table 1.

### 4.10. Electrophoretic Mobility Shift Assay 

To perform EMSA analyses, the dsDNA probe corresponding to G4FS was obtained by mixing equal amounts of each single-stranded oligodeoxynucleotide in a 150 mM NaCl solution. After incubation at 90 °C for 5 min, the solution was allowed to cool down slowly to room temperature. The duplex was resolved in a nondenaturing 20% polyacrylamide gel, visualized using UV shadowing and purified from the gel. DNA concentration was measured as stated in Section 4.3 of Materials and Methods.

dsDNA, ssDNA and RNA probes (200 ng) were 5’-end-labeled with (γ-^32^P)-ATP (3000 Ci/mmol) (Perkin Elmer, Madrid, Spain) using T4 polynucleotide kinase (New England BioLabs, MA, USA) in a 10 µL reaction mixture, according to the manufacturer’s instructions. After incubation at 37 °C for 1 h, 90 µL of Tris-EDTA buffer (1 mM EDTA and 10 mM Tris, pH 8.0; Sigma-Aldrich, Madrid, Spain) was added to the reaction mixture, which was subsequently filtered through a Sephadex G-25 (Sigma-Aldrich, Madrid, Spain) spin-column to eliminate the unincorporated (γ-^32^P)-ATP.

Radio-labeled probes (100,000 cpm, [γ-32P]-ATP) were incubated with either HeLa nuclear protein extracts, purified TYMS or HpTYMS-G4-T PPRH. Both BSA and DHFR proteins were used as negative controls. Poly(dI:dC) (3 µg) was added to each reaction as an unspecific competitor. Binding reactions were performed in the presence of a binding buffer (5% glycerol, 0.5 mM DTT, 4 mM MgCl_2_, 36 mM KCl, 0.5 mM EDTA, 25 mM Tris-HCl, pH 8.0; all reagents were purchased from Sigma-Aldrich). The products of the binding reactions were electrophoretically resolved in 5% polyacrylamide and 5% glycerol native gels in 0.5× TBE buffer, at a fixed voltage of 220 V and 4 °C. Gels were dried at 80 °C and scanned on a Storm 840 PhosphorImager (Molecular Dynamics, GE Healthcare Life Sciences, Barcelona, Spain). ImageQuant software v5.2 was used to visualize and quantify the results (GE Healthcare, Barcelona, Spain).

### 4.11. Detection of G4 Structures with ThT upon DNA Binding Assays

To detect G4 structures after DNA binding assays, 1.5 µg of each oligonucleotide, alone or combined with the indicated oligonucleotide, was incubated at 90 °C for 5 min in water, then diluted with a buffer containing 100 mM KCl and 100 mM Tris/HCl (pH 7.5) in a final volume of 100 µL and slowly cooled to room temperature (2 h). 

Samples (75 µL) were loaded on nondenaturing 12% polyacrylamide gels (10 cm) containing 10 mM KCl in 1× TBE buffer and electrophoresed for 1–2 h at 150 V. After electrophoresis, gels were stained with 5 µM of ThT solution for 15 min under agitation, briefly washed in water, and exposed to a UV light lamp to obtain images. Then, the same gels were further stained with 1 µg/mL ethidium bromide solution for 10 min under agitation and washed for 10 min in water. Images were captured under a UV light lamp or using the Gel Doc^TM^ EZ with the Image Lab Software, Version 6.0.

### 4.12. Cell Culture

HeLa and PC-3 cell lines obtained from the cell bank resources from University of Barcelona were grown in Ham’s F12 medium supplemented with 10% fetal bovine serum (GIBCO, Invitrogen, Barcelona, Spain) or in Roswell Park Memorial Institute medium (RPMI), which is deficient in thymidine, supplemented with 7% dialyzed fetal bovine serum and incubated at 37 °C in a humidified 5% CO2 atmosphere.

### 4.13. Transfection of the PPRH

One day before transfection, cells were plated in 6-well dishes in 800 µL of either F12 or RPMI medium. The transfection consisted of mixing N-[1-(1,2-Di-(9Z-octadecenoyl)-3-trimethylammoniumpropane methyl sulfate (DOTAP; Biontex, Germany) with the PPRH and serum-free medium in volumes of up to 200 μL. Unless stated otherwise, the molar ratio of PPRH/DOTAP was 1:100 (100 nM/10 µM). After 20 min of incubation at room temperature, the mixture was added to the cells to attain a final volume of 1 mL. 

### 4.14. Western Blot Analyses

Total protein extracts from HeLa cells (30,000) were obtained using 100 μL of RIPA buffer (1% Igepal, 0.5% sodium deoxycholate, 0.1% SDS, 150 mM NaCl, 1 mM EDTA, 1 mM PMSF, 10 mM NaF and 50 mM Tris-HCl, pH 8, containing additionally the Protease inhibitor cocktail (P8340-5ML); all the above were purchased from Sigma Aldrich, Madrid, Spain, but Tris-HCl was from PanReac AppliChem, Barcelona, Spain). Extracts were incubated for 5 min at 4 °C and cell debris was removed by centrifugation (16,300× *g* at 4 °C for 10 min). Protein concentrations were determined using a Bio-Rad protein assay based on the Bradford method and using bovine serum albumin as a standard.

Protein extracts were electrophoresed in 10% SDS-polyacrylamide gels and transferred to polyvinylidene difluoride membranes. Blocking of membranes was performed using 5% Blotto. In the case of primary antibody against TYMS (1:100 dilution; sc-376161, Santa Cruz Biotechnology, Heidelberg, Germany), membranes were probed overnight at 4 °C, whereas primary antibody against α-Tubulin (1:100 dilution; CP06, Merck, Darmstadt, Germany) membranes were probed for 90 min at room temperature. Signals of both proteins were detected by secondary horseradish peroxidase-conjugated anti-mouse antibody (1:2500 dilution; sc-516102, Santa Cruz Biotechnology, Heidelberg, Germany). Chemiluminescence was detected with the ImageQuant LAS 4000 mini imager (GE Healthcare, Barcelona, Spain). Quantification was performed using the ImageQuant 5.2 software.

### 4.15. Combination Treatment of 5-FU plus HpTYMS-G4-T

Cells were plated in 6-well dishes in RPMI medium one day before transfection or 5-FU treatment. The 5-FU (Sigma-Aldrich, Madrid, Spain) was prepared from powder as a 100 mM stock solution in DMSO and diluted in RPMI medium. The transfection was performed as indicated in the Section 4.13, Transfection. Cells were incubated with 5-FU alone or combined concurrently with the PPRH in a final volume of 1 mL. 

### 4.16. MTT Assay

Cells (10,000) were plated in 6-well dishes in F12 medium or RPMI medium. Five days after transfection or 5-FU treatment, 3-(4,5- dimethylthiazol-2-yl)-2,5-diphenyltetrazolium bromide and sodium succinate (both from Sigma-Aldrich, Madrid, Spain) were added to the culture medium (final concentration 0.63 mM and 100 μM, respectively) and incubated for 2.5 h at 37 °C. Then, the culture medium was removed and the lysis solution (0.57% of acetic acid and 10% of SDS in DMSO) (Sigma-Aldrich, Madrid, Spain) was added. Absorbance was measured at 570 nm in a Modulus Microplate spectrophotometer (Turner BioSystems, Madrid, Spain). Cell viability results were expressed as the percentage of cell survival relative to the controls.

### 4.17. Statistical Analyses

Statistical analyses were performed using GraphPad Prism 6 (GraphPad Software, CA, USA). All data are shown as the mean ± SEM of at least three independent experiments. The levels of statistical significance were denoted as follows: *p* < 0.05 (*), *p* < 0.01 (**), *p* < 0.001 (***) or *p* < 0.0001 (****). 

## 5. Conclusions

In this work, we identified a G4FS in the 5’UTR of the *TYMS* gene and confirmed its folding into a G4 structure in both DNA and RNA backbones. Additionally, we determined that TYMS protein directly interacts with G4FS either as dsDNA, ssDNA or RNA, which may be involved in transcriptional and translational autoregulation of TYMS expression. Finally, the HpTYMS-G4-T PPRH targeting the complementary strand of the G4FS decreased both TYMS mRNA and protein levels in cancer cells, leading to cell death and showing a synergic effect in combination with 5-FU.

## Figures and Tables

**Table 1:** Oligonucleotides used in this study.

Name	Sequence (5’-3’)	Length	Assay
5’UTR-TYMS-Fw	GAGCAGGAAGAGGCGGAGCG	20	PCR
5’UTR-TYMS-Rv	GCAGCTCCGAGCCGGCCACAGG	22	PCR
APRT-Ex1-Fw	CACCCCAGGCGTGGTATTCA	20	PCR
APRT-Ex2-Rv	CTGCGATGTAGTCGATGCGG	20	PCR
TYMS-Fw	CCTCGGTGTGCCTTTCAACATC	22	qPCR
TYMS-Rv	GGTCTGGGTTCTCGCTGAAGC	21	qPCR
PPIB-Fw	GGAGATGGCACAGGAGGAAA	20	qPCR
PPIB-Rv	CGTAGTGCTTCAGTTTGAAGTTCTCA	26	qPCR
RNA-G4	GGGAAAAGGCGCGCGGAAGGGG	22	EMSA, CD, UV, Flu
ssDNA-G4-Fw-E	GGGAAAAGGCGCGCGGAAGGGG	22	EMSA
ssDNA-G4-Rv-E	CCCCTTCCGCGCGCCTTTTCCC	22	EMSA
ssDNA-G4-Fw	CGGGAAAAGGCGCGCGGAAGGGGT	24	TGS, CD, UV, Flu, NMR
ssDNA-G4-Rv	ACCCCTTCCGCGCGCCTTTTCCCG	24	TGS

PCR (polymerase chain reaction), qPCR (quantitative PCR), EMSA (electrophoretic mobility shift assay), CD (circular dichroism), UV (UV absorbance spectroscopy), Flu (thioflavin T fluorescence spectroscopy), Thioflavin T gel staining (TGS), NMR (nuclear magnetic resonance).

## Supplementary Materials

Supplementary materials can be found at https://www.mdpi.com/1422-0067/21/14/5028/s1.

## Abbreviations

TYMS	Thymidylate synthase
5-FU	5-fluorouracil
ASO	Antisense oligodeoxynucleotides
G4	G-quadruplex structures
G4FS	G4 forming sequence
PPRH	Polypurine reverse Hoogsteen hairpins
ThT	Thioflavin T
DOTAP	N-(1-(1,2-Di-(9Z-octadecenoyl)-3-trimethylammoniumpropane methyl sulfate
