# Supplementary material for: Detection of a G-Quadruplex as a Regulatory Element in Thymidylate synthase for Gene Silencing Using Polypurine Reverse Hoogsteen Hairpins
Source: Int J Mol Sci. 2020 Jul 16;21(14):5028. doi: 10.3390/ijms21145028 (PMC7404261; doi:10.3390/ijms21145028)
Supplement: Supplementary file 1 [file ijms-21-05028-s001.pdf]

Supplementary data

A)

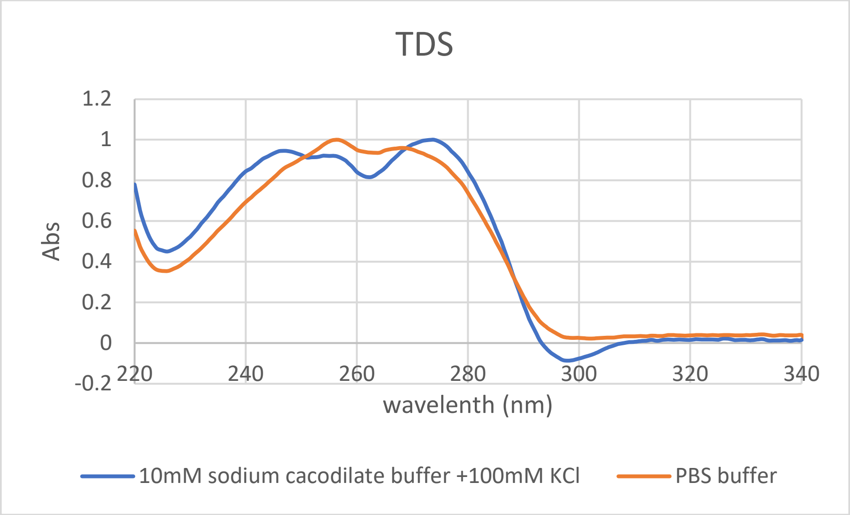

B)

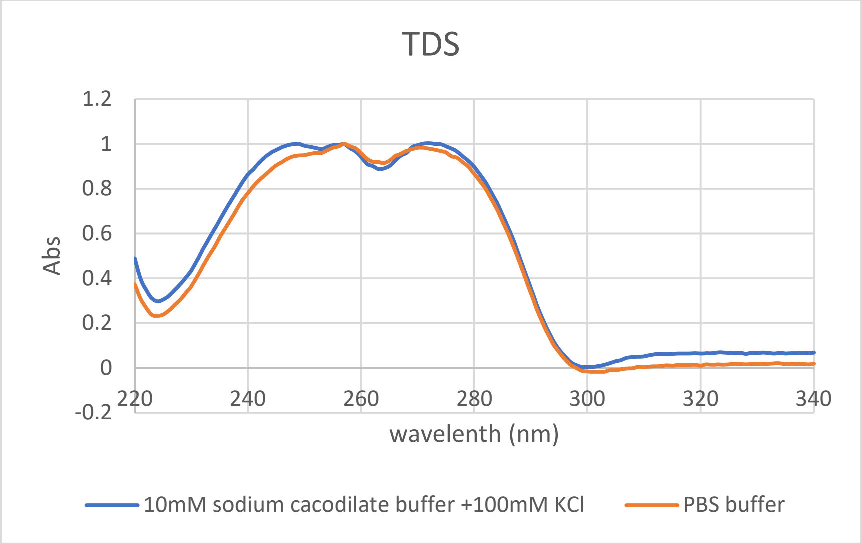

Figure S1: Thermal Differentiation Spectra of ssDNA-G4-Fw (A) and RNA-G4 (B) in two different buffers.

A)

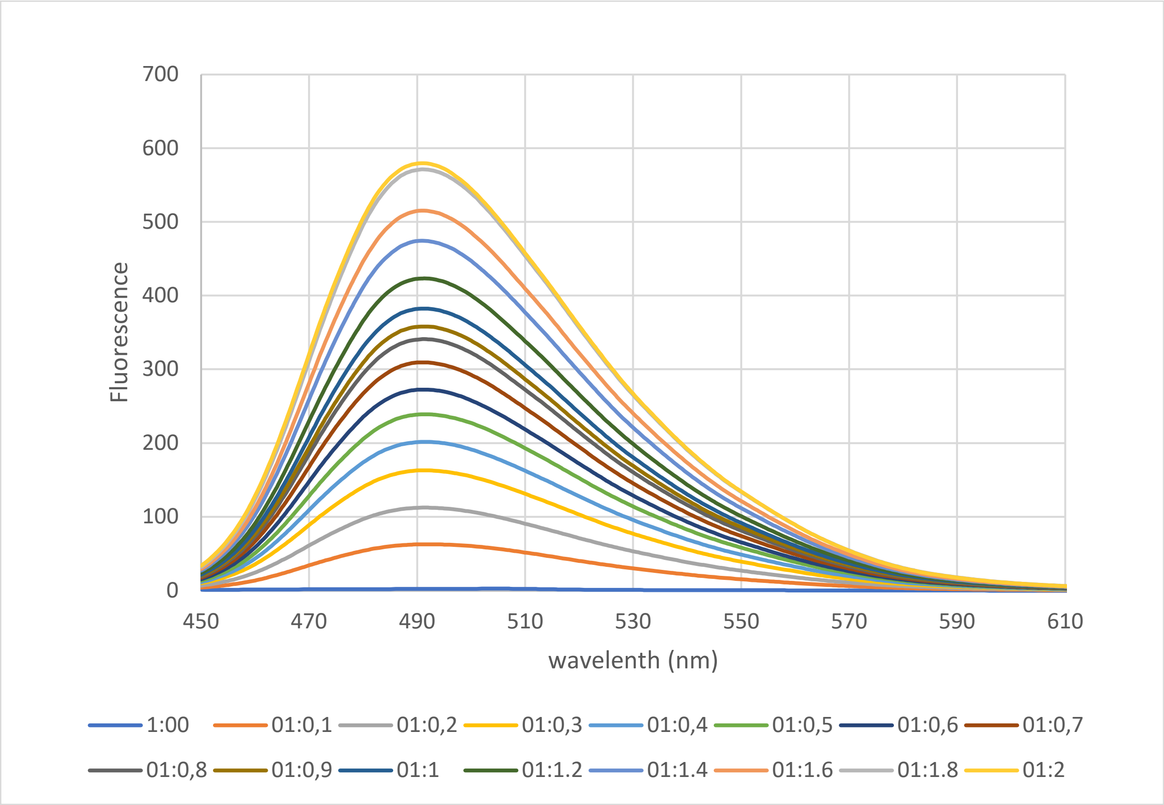

B)

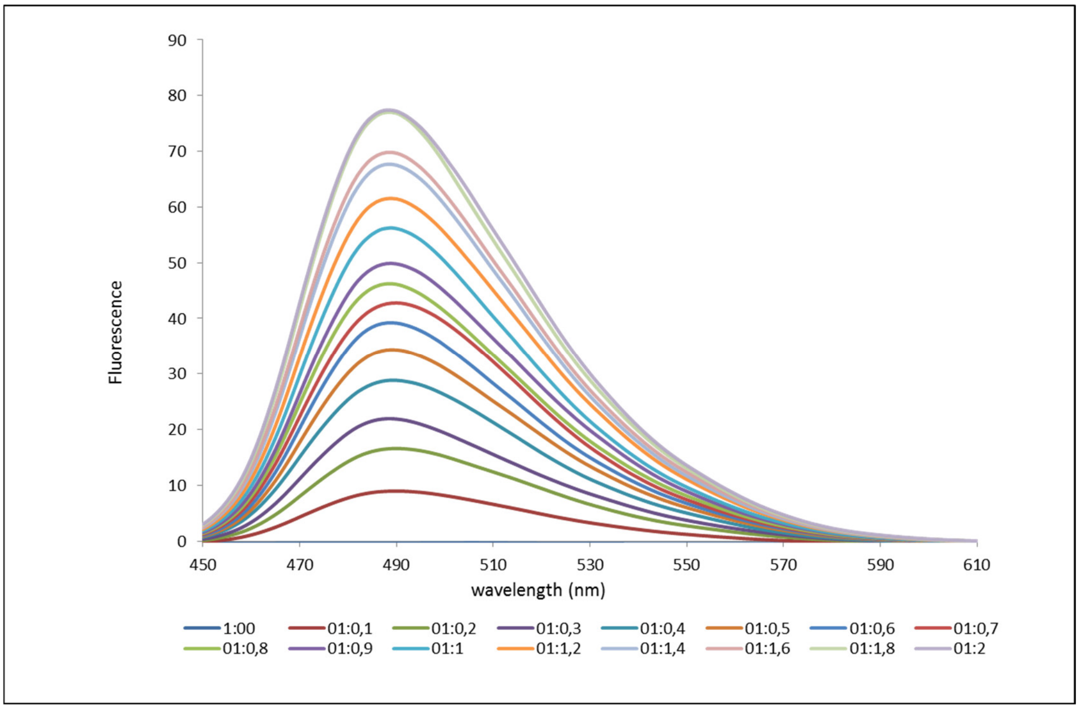

Figure S2: Fluorescence spectra of ThT with increasing concentrations of ssDNA-G4-Fw (A) and RNA-G4 (B) ODN. Initial 1: 0 (3μM ThT: 0 ODN). Final 1: 2 (3μM ThT: 6μM ODN).

The data were fitted according to a 1:1 binding model. The following equation was used to calculate the association constant (K_a)

$$\Delta Fcalc = \left(\frac{\Delta F_{max}}{2C_t}\right)\left[\left\{[Q_o] + [C_o] + \frac{1}{K_a}\right\} - \left\{\sqrt{\left([Q_o] + [C_o] + \frac{1}{K_a}\right)^2 - 4[Q_o][C_o]}\right\}\right]$$

$[Q_o]$ is the concentration of the oligonucleotide, $[C_o]$ and C_t are the concentrations of the initial free and final ligand, respectively. Finally, ΔF_{max} corresponds to the maximum increment of fluorescence. Note that the model (ODN + ligand → complex) assumes two states, and the fluorescence is the sum of the free and the complex ligand.

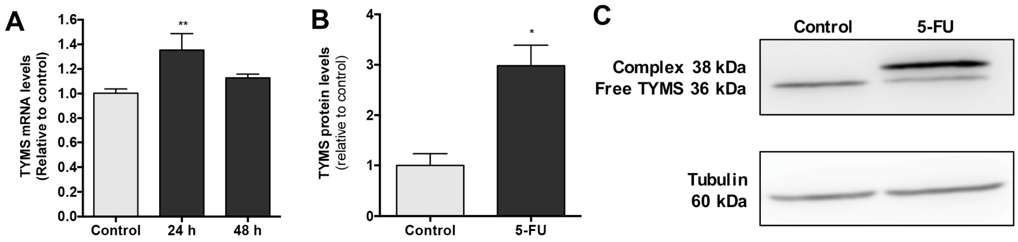

Figure S3: Effect of 5-FU on the levels of TYMS mRNA and protein. Hela cells (30,000) were incubated with 3 μM of 5-FU in RPMI medium. (A) TYMS mRNA levels were determined by RT-qPCR 24 h and 48 h after 5-FU treatment. Cyclophilin B (PPIB) was used to normalize the results. Statistical significance was determined using one-way ANOVA with Dunnett's multiple comparisons test (**p <0.01). (B) Total TYMS protein levels on HeLa cells after 24h of treatment. The total amount of TYMS protein levels were quantified (free TYMS protein, corresponding to 36 kDa band, plus the ternary complex FdUMP-TYMS-mTHF, corresponding to 38 kDa band). Statistical significance was determined using an Unpaired Student's T test (*p < 0.05). Tubulin protein levels were used to normalize the results. (C) Representative images of Western blots.
